# Effect of Annealing Time on the Cyclic Characteristics of Ceramic Oxide Thin Film Thermocouples

**DOI:** 10.3390/mi13111970

**Published:** 2022-11-13

**Authors:** Yuning Han, Yong Ruan, Meixia Xue, Yu Wu, Meng Shi, Zhiqiang Song, Yuankai Zhou, Jiao Teng

**Affiliations:** 1 Department of Electronic Information, Beijing Information Science and Technology University, Beijing 100192, China; 2 Department of Precision Instruments, Tsinghua University, Beijing 100084, China; 3 Department of Materials Physics and Chemistry, University of Science and Technology Beijing, Beijing 100083, China; 4 Qiyuan Laboratory, Beijing 100094, China; 5 MEMS Institute of Zibo National High-Tech Industrial Development Zone, Zibo 255000, China

**Keywords:** thin film thermocouples, temperature, anneal

## Abstract

Oxide thin film thermocouples (TFTCs) are widely used in high-temperature environment measurements and have the advantages of good stability and high thermoelectric voltage. However, different annealing processes affect the performance of TFTCs. This paper studied the impact of different annealing times on the cyclic characteristics of ceramic oxide thin film thermocouples. ITO/In_2_O_3_ TFTCs were prepared on alumina ceramics by a screen printing method, and the samples were annealed at different times. The microstructure of the ITO film was studied by scanning electron microscopy (SEM), X-ray diffraction (XRD), and X-ray photoelectron spectroscopy (XPS). The results show that when the annealing temperature is fixed, the stability of the thermocouple is worst when it is annealed for 2 h. Extending the annealing time can improve the properties of the film, increase the density, slow down oxidation, and enhance the thermal stability of the thermocouple. The thermal cycle test results show that the sample can reach five temperature rise and fall cycles, more than 50 h, and can meet the needs of stable measurement in high temperature and harsh environments.

## 1. Introduction

With the development of microelectromechanical system (MEMS) technology, the technology of using high-temperature sensors to measure the temperature of target pipelines has emerged [1,2,3,4,5,6]. For example, the working temperature of large-scale equipment such as iron and steel smelting, casting high temperature, and gas turbines is generally 600–1100 °C, of which the temperature of the exhaust flue is about 25–800 °C [7,8,9]. Monitoring the high temperature generated by the exhaust flue can judge the process production status, which is conducive to ensuring safe and effective production and research. In traditional technology, infrared radiation temperature measurement has been adopted, but the infrared temperature measurement requires that there is no barrier between the infrared probe and the surface to be measured of the exhaust gas flue, and that the surface to be measured is required to be free of contaminants and other impurities. However, it is difficult to ensure that the surface to be measured is free of pollutants in the actual industrial environment, which leads to low accuracy of the temperature of the monitored target pipeline.

Thermocouples can be classified into two categories: metal type and ceramic type. The traditional K-type and S-type thin film thermocouples do not meet the requirements of such a high-temperature test because they are easy to oxidize and the change of microstructure reduces their temperature measurement accuracy and life span. The S-type (Pt/Pt-10% Rh) thin film thermocouple is expensive, and the thermoelectric potential of the positive element decreases when working at 600~800 °C [10]. Metal thermocouples such as the Pt/Pd film thermocouple, with the progress of the thermal cycle, the microstructure changes such as the growth of pores and grains in the film lead to the decrease of thermoelectric potential, and the destruction of the electrode film structure will lead to the failure of the film thermocouple. Therefore, the stability of the film structure plays a crucial role in preventing the failure of the thin film thermocouple during the thermal cycling process [11]. On the contrary, thermocouples made of In_2_O_3_ and ITO (In_2_O_3_:SnO_2_ = 90:10 wt%) show higher thermoelectric voltage and better thermal stability [4,12,13,14,15]. The annealing process will also affect the performance of the ITO/In_2_O_3_ thin film thermocouple. When annealing under N^2^ at 500 °C, the thermoelectric response will be unstable when working at high temperatures for a long time, while the thermocouple annealed in air at 1000 °C shows better stability [16]. ITO is a typical oxide semiconductor, and annealing has a significant effect on its thermoelectric properties, especially the Seebeck coefficient and thermoelectric stability [7]. In the process of preparing ITO/In_2_O_3_ thin film thermocouples (TFTCs), it is found that the test results of the thermoelectric cycle cannot meet the needs. The linearity of the relationship curve between temperature and voltage is poor, but the linearity and repeatability are improved after several cycles; this phenomenon will make the sensor temperature test inaccurate and limit its application. We want to improve this phenomenon by improving the annealing process so that ITO/In_2_O_3_ TFTCs have a stable performance in the test process.

In this paper, ITO/In_2_O_3_ TFTCs were prepared on alumina ceramics by screen printing [17,18]. In contrast, ITO TFTCs annealed for different annealing times were studied accordingly. Different samples are made at 1000 °C for 1 h, 2 h, 3 h, and 5 h, respectively. The microstructure of the ITO film was studied by scanning electron microscopy (SEM), X-ray diffraction (XRD), and X-ray photoelectron spectroscopy (XPS). The results show that when the annealing temperature is fixed, the stability of the thermocouple is worst when it is annealed for 2 h. When the annealing time is above 3 h, the thermocouple can maintain stability and excellent linearity. The experimental phenomenon shows that extending the annealing time can improve the properties of the film, increase the density, slow down oxidation, and enhance the thermal stability of the thermocouple. The thermal cycle test can reach five temperature rise and fall cycles, and the time exceeds 50 h. Extending the high-temperature annealing time is conducive to the stability of the thermoelectric output of the thermocouple.

## 2. Materials and Methods

### 2.1. Fabrication of ITO/In_2_O_3_ Thin Film Thermocouple

**Figure 1:**
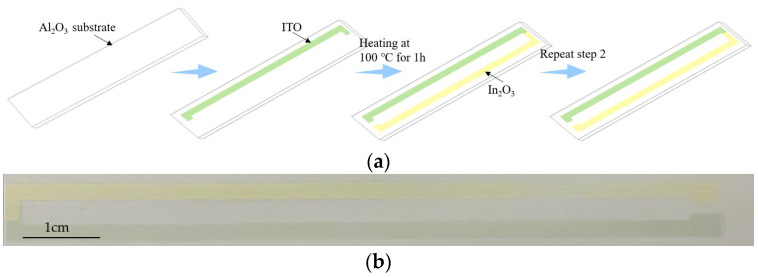
(**a**) Fabrication process of ITO/In_2_O_3_ TFTCs by screen-printing. (**b**) Optical image of the ITO/In_2_O_3_ thermocouple.

ITO/In_2_O_3_ TFTCs were prepared by a screen printing process. The substrate is 99 alumina ceramic sheets (150 mm × 15 mm × 1 mm). The alumina ceramic sheet was fixed under the graphical screen printing plate (20 cm × 30 cm), which is 300 meshes; each electrode is 30–35 cm long and 0.2 cm wide. The scraper is made of rubber with a width of 5 cm. The fabrication process of ITO/In_2_O_3_ TFTCs by screen-printing is shown in Figure 1a. When printing, first of all, the position of the scraper needs to be adjusted to keep the scraper at a 45° angle with the printing plane, apply pressure to the screen printing plate with constant force and uniform speed, and squeeze the prepared ITO slurry onto the alumina ceramic substrate [19]. The ITO electrode is solidified after being heated at 100 °C for an hour in a furnace; then the In_2_O_3_ slurry is printed on the substrate by the same steps. The positive electrode and negative electrode of the thermocouple are completed. After being annealed at 800 °C for an hour, the stress in the film is released. Figure 1b shows the optical image of the ITO/In_2_O_3_ thermocouple. ITO/In_2_O_3_ thin film was then annealed in air at 1000 °C at different times. Different samples are made at 1000 °C for 1 h, 2 h, 3 h, and 5 h, respectively. ITO/In_2_O_3_ TFTCs were made. A copper wire (20 cm) was coated on the cold junction of ITO and In_2_O_3_ with silver paste and dried at 150 °C for 2 h.

### 2.2. Measurements

Surface images were obtained by SEM. Using XRD, we analyzed the crystal structure of ITO and In_2_O_3_ films; the step was 0.02°. The chemical composition and chemical state were analyzed by XPS.

ITO/In_2_O_3_ TFTCs were statically calibrated with a standard calibration furnace. The standard S-type thermocouple is used to continuously monitor the furnace temperature, that is, the hot junction temperature and the platinum resistance is used to measure the cold junction temperature. ITO/In_2_O_3_ TFTCs were calibrated with the temperature of hot junction up to 1050 °C.

## 3. Results

### Microstructures of ITO and In_2_O_3_ Films

**Figure 2:**
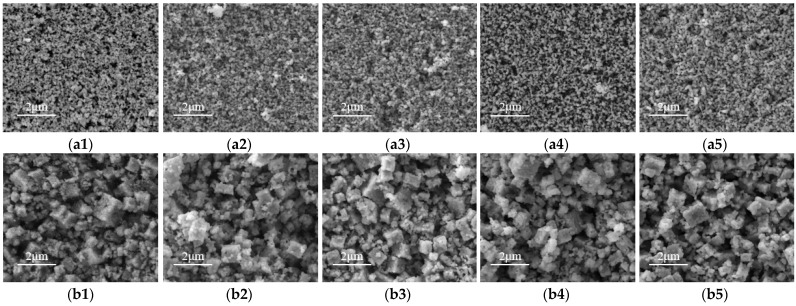
Surface morphology of the ITO ((**a1**) as-deposited; (**a2**) 1 h; (**a3**) 2 h; (**a4**) 3 h; (**a5**) 5 h), and In_2_O_3_ ((**b1**): as-deposited; (**b2**) 1 h; (**b3**) 2 h; (**b4**) 3 h; (**b5**) 5 h) films annealed for different annealing times.

SEM micrographs of ITO and In_2_O_3_ films treated for different annealing times are shown in Figure 2.

**Figure 3:**
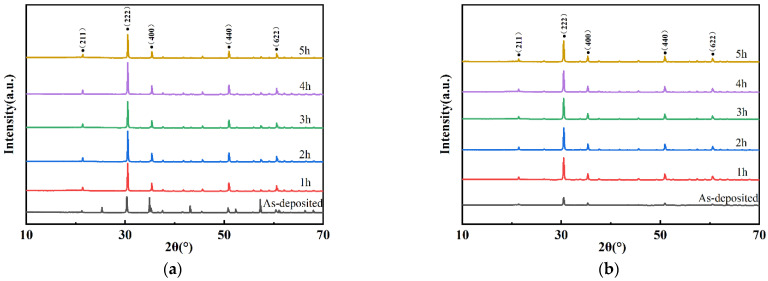
XRD patterns of films annealed for different annealing times: (**a**) ITO; (**b**) In_2_O_3_.

**Figure 4:**
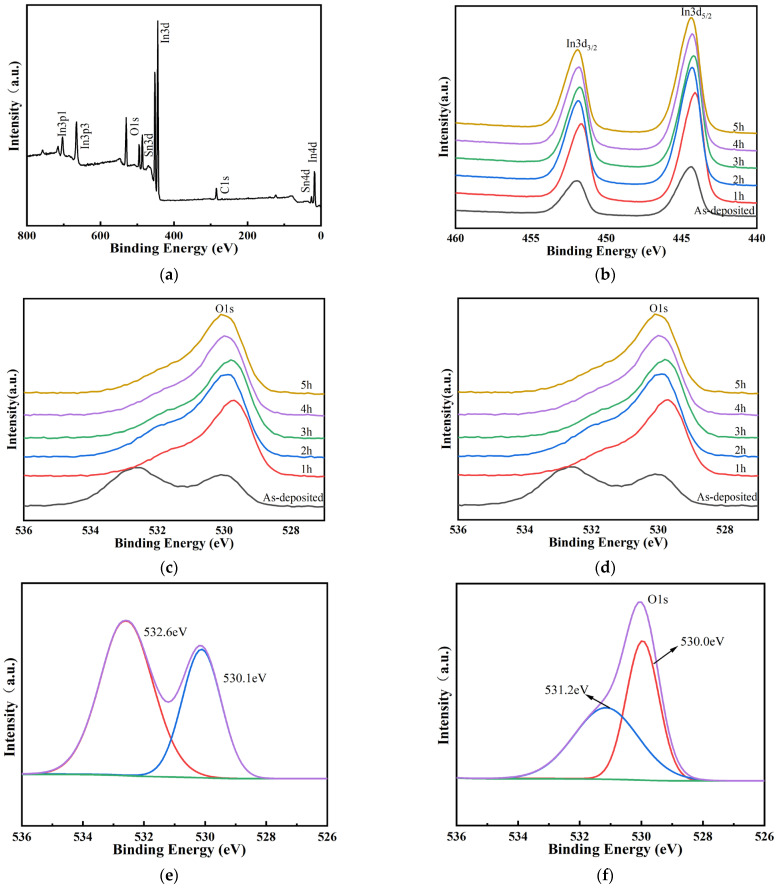
XPS of the ITO thin film: (**a**) full-spectra (**b**–**d**) XPS high-resolution peaks of In 3d, Sn 3d, and O1s, respectively; (**e**,**f**) XPS high-resolution peaks of O1s of as-deposited and 1 h.

The grain size of ITO and In_2_O_3_ films increases with the prolongation of heat treatment time. As can be seen from Figure 3, the surface morphology of the films changed slightly with the extension of heat treatment time, and all the films showed a similar density. It can be seen from Figure 4 that the In_2_O_3_ thin films are all cubic particles after annealing at different times, that is, the cubic shapes of In_2_O_3_. The grain-sized In_2_O_3_ films become denser with the prolongation of heat treatment time. This is because the more oxygen vacancies are occupied in the In_2_O_3_ film, the more sufficient the oxidation is. With the prolongation of annealing time, the space available for oxygen gradually decreases. Therefore, with the prolongation of heat treatment time, the film density changes slowly [20,21].

Figure 3 shows XRD patterns of ITO and In_2_O_3_ thin films for different annealing times. Figure 3a shows the as-deposited ITO film was amorphous. After being annealed at 1000 °C, there are diffraction peaks at 21.4°, 30.5°, 35.4°, 37.6°, 45.6°, 51.0°, and 60.6°, which correspond to (211), (222), (400), (440), and (622) of cubic bixbyite structure In_2_O_3_. The preferred growth direction of all ITO films is the (400) [22,23]. With the increase of annealing time, the diffraction peak corresponding to the (222) crystal plane of In_2_O_3_ shifts to a low angle. This is because high-temperature calcination leads to lattice expansion and an increase in crystal plane spacing, which makes the diffraction angle shift [24]. The crystal phase characteristic peaks of Sn are not shown in the figure, which means that Sn atoms replace the In atoms in the In_2_O_3_ crystal lattice [25,26,27,28], resulting in better sensitivity of the sensor [29].

The surface composition and elemental oxidation states of ITO thin film were investigated by XPS analysis. Figure 4a is the XPS full spectrum of the ITO film heat-treated at 1000 °C for 5 h, and the nuclear energy levels of In3d, Sn3d, and O1s were analyzed. Figure 4b shows that the peak of In 3d_5/2_ at 444.5 eV indicates the incorporation of In3+ in In_2_O_3_. As shown in Figure 4c, the two peaks at 486.5 eV and 495.0 eV were ascribed to Sn 3d_5/2_ and Sn 3d_3/2_, respectively, which are characteristic of Sn4+. The peak at 486.4 eV belongs to the binding energy of Sn 3d_5/2_, which corresponds to the Sn4+ bonding state in the Sn4+-bonded ITO lattice. Figure 4d shows that there were two peaks in the energy level of O, and the peak fitting was performed on the as-deposited and annealed for 5 h, as shown in Figure 4e,f. It can be seen from Figure 4f that O1s had two binding energy peaks, 530.0 and 531.2 eV, respectively, which corresponded to oxygen in the In-O bond and oxygen in the Sn-O bond [30].

**Figure 5:**
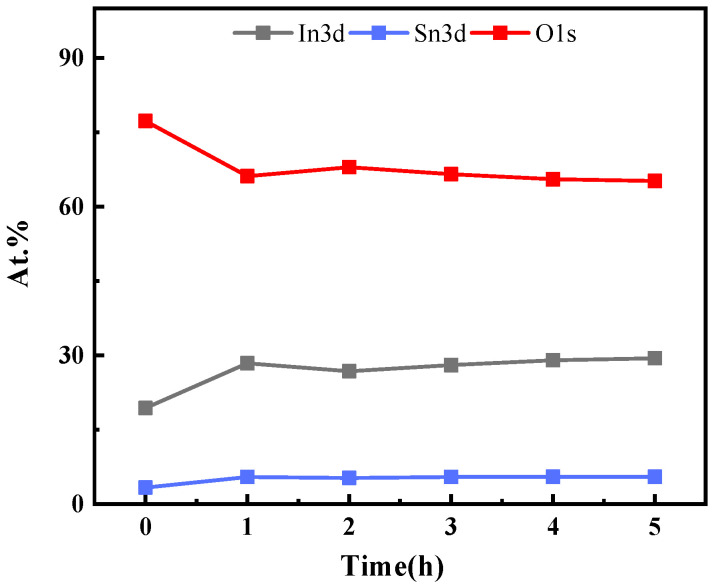
XPS results of ITO films fabricated for different annealing times.

Using the semi-quantitative fitting method, it was found that the atomic ratio of In_2_O_3_/SnO_2_ was 9:1. XPS analysis showed the bonding state of In3+ and Sn4+ in the ITO film Figure 5 shows the XPS results of ITO films prepared at different annealing times. Compared with the as-deposited films, the oxygen atom ratio decreases after annealing at 1000 °C. With the increase of annealing time, the atomic ratios of O, In and Sn remain relatively stable.

Table 1 lists the conductivity of ITO and In_2_O_3_ films after annealing at different times. The conductivity increased with the prolongation of heat treatment time, which made the scattering of charge carriers decrease with the increase in grain size [15]. When the ITO film was annealed at 1000 °C for 5 h, its conductivity reached 3.82 × 10^3^ S/m, which was 1.59 times that of the as-deposited sample. Therefore, the electrical conductivity of ITO films will be affected by the annealing time.

**Figure 6:**
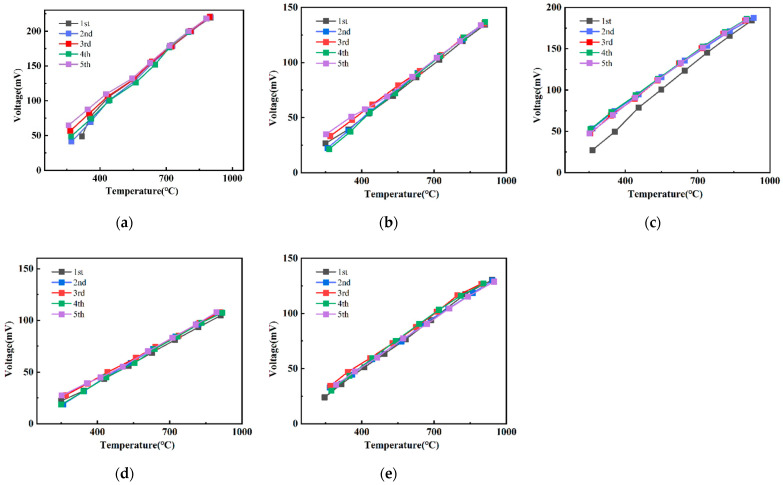
Thermoelectric output for different annealing times: (**a**) as-deposited; (**b**) 1 h; (**c**) 2 h; (**d**) 3 h; (**e**) 5 h.

Figure 6 shows the thermoelectric output of the ITO/In_2_O_3_ TFTC from room temperature to 1150 °C. The thermal output of the thermocouple is stable over five thermal cycles and can be used over and over again. The stability of the thermocouple is the worst when it is annealed for 2 h in Figure 6b. As shown in Figure 6, ITO/In_2_O_3_ TFTC continues to work after five temperature rise and fall cycles, about 50 h, and the linearity is still high.

**Figure 7:**
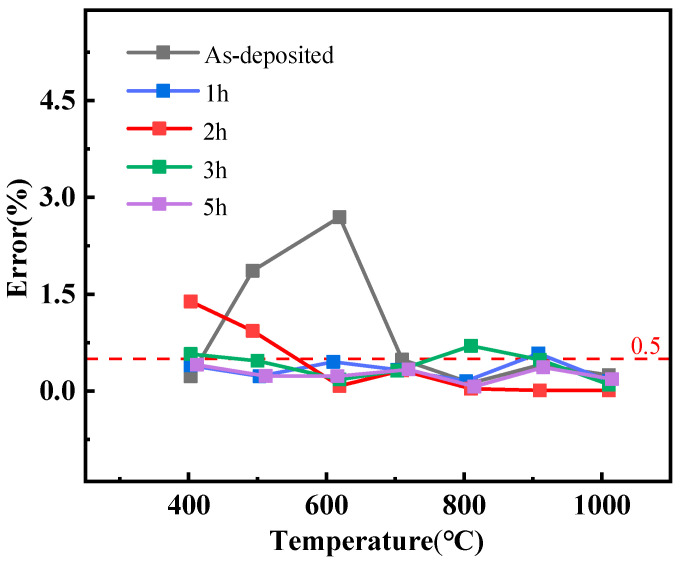
Errors of ITO/In_2_O_3_ TFTCs annealed for different times.

A third term polynomial is used to better describe the thermoelectric potential versus temperature in Equation (1):

V(∆T) = A(∆T)^3^ + B(∆T)^2^ + C(∆T) + D, (1)

the fitting results are listed in Table 2. The fitting correlation coefficients (R^2^) of all calibration curves are greater than 0.999. The coefficients of the third and second polynomials are smaller, indicating that the curve is nearly linear. According to the fitting results, the average Seebeck coefficient of ITO/In_2_O_3_ TFTCs that were annealed for five hours is 148.62 μV/°C. At the same time, the Seebeck coefficient of the thermocouple decreases with the annealing time of 3 h, which is attributed to the increase in carrier concentration [7]. We used the data in Table 2 as the standard temperature measurement expression of four groups of thin-film thermocouples to calculate the temperature deviation between the third calibration result and the expression. The result is shown in Figure 7; the expression of error is where ΔT is the temperature difference and ΔT1 is the temperature difference value calculated by the formula in Table 2. The temperature measurement errors of the unannealed TFTCs reached 2.69%, which is very undesirable. However, the maximum temperature measurement errors of films annealed for 1 h, 2 h, 3 h, and 5 h are 0.58%, 1.39%, 0.70%, and 0.41%, respectively, and the temperature measurement errors of annealed for 5 h are not more than 0.50%.

## 4. Conclusions

ITO/In_2_O_3_ TFTCs were prepared on alumina ceramics by screen printing. TFTCs annealed at different times were studied. The results show that when the annealing temperature is fixed, the stability of the thermocouple is the worst when it is annealed for 2 h. Extending the annealing time can improve the properties of the film, increase the density, slow down oxidation, and enhance the thermal stability of the thermocouple. The thermal cycle test results show that the sample can reach five temperature rise and fall cycles, more than 50 h.

## Figures and Tables

**Table 1:** The conductivity of ITO and In_2_O_3_ annealed at different times.

	Annealing Time (h)	0	1	2	3	5
Conductivity (S/m)	ITO	2.41 × 10^3^	2.9 × 10^3^	3.25 × 10^3^	3.48 × 10^3^	3.82 × 10^3^
	In_2_O_3_	12.2	15.5	17.3	23.9	52.9

**Table 2:** Thermoelectric response and fitting results of ITO/In_2_O_3_ TFTCs for different annealing times.

Annealing Time (h)	V(ΔT) = A(ΔT)^3^ + B(ΔT)^2^ + C(ΔT) + D				Correlation Coefficient (R^2^)	Average Seebeck Coefficient (µV/°C)
	A (mV/°C^3^)	B (mV/°C^2^)	C (mV/°C)	D (mV)		
0	7.52 × 10^−8^	−1.54 × 10^−5^	0.36	−13.04	0.99914	256.65
1	1.70 × 10^−8^	−3.22 × 10^−5^	0.18	−13.04	0.99981	155.29
2	−7.27 × 10^−8^	7.17 × 10^−5^	0.21	−8.89	0.99979	209.45
3	4.21 × 10^−8^	−7.14 × 10^−5^	0.16	−11.73	0.99979	123.75
5	−5.75 × 10^−8^	5.80 × 10^−5^	0.15	−14.27	0.99993	148.62

## Data Availability

Not applicable.
